# Hemodynamic Effects of Interfacility Dexmedetomidine Infusions During Transport of Patients with Alpha-2-adrenergic Agonist Withdrawal

**DOI:** 10.5811/westjem.62953

**Published:** 2026-09-09

**Authors:** Scott Goldstein, Nathaniel Zahustecher, Derek Isenberg, Eric Rosen, Celine Bennett

**Affiliations:** * Temple University Hospital, Department of Emergency Medicine, Division of Emergency Medical Services, Philadelphia, Pennsylvania; † Lewis Katz School of Medicine at Temple University, Philadelphia, Pennsylvania; ‡ Temple Transport Team, Philadelphia, Pennsylvania

## Abstract

**Background:**

In the Philadelphia region, there has been an increase in the potency of illicit opioids as well as the addition of nonopioid adulterants. Currently, medetomidine, a veterinary anesthetic that acts as an alpha-2-adrenergic agonist, is a common adulterant in the illicit opioid supply, causing both unique overdose and withdrawal syndromes. Although opioid withdrawal alone is not deadly, with added adulterants, especially medetomidine, this is no longer the case. Dexmedetomidine is an alpha-2-adrenergic agonist frequently used for sedation in the emergency department and intensive care unit. Dexmedetomidine is also used off-label to treat the life-threatening withdrawal from alpha-2-agonists.

**Objective:**

This study describes the dosing and hemodynamic effects associated with the interfacility administration of dexmedetomidine in patients with alpha-2 adrenergic agonist withdrawal. Secondarily, we describe the safety of dexmedetomidine in this patient population.

**Methods:**

We conducted a retrospective case review of patients receiving dexmedetomidine infusions in the interfacility setting for presumed alpha-2–adrenergic agonist withdrawal. Demographic data, dosing, and vital signs were analyzed descriptively.

**Results:**

Over a six-month period, of 217 cases identified, 182 contained complete hemodynamic data. The average age was 39.5 years, and 67% were male. The average transport time was 15.7 minutes (range, 5–40 minutes). The mean dexmedetomidine infusion rate was 1.32 (0.31) mcg/kg/hr. The average mean arterial pressure decreased from 122.1 mm Hg (21.7) at the time of initial patient contact to 118.3 mm Hg (21.7) (mean difference, 3.80; 95% CI, 1.96–5.64) at final patient interaction. Heart rate also showed a minimal change from 104.9 (23.7) to 103.6 (21.8) beats/min (mean 1.3; 95% CI, −0.48 to 3.08). No cases of severe hypotension or clinically significant bradycardia requiring intervention were documented (one-sided 95% CI, 0–1.7%).

**Conclusion:**

In this case series of patients, prehospital dexmedetomidine infusions to treat alpha-2-agonist withdrawal were associated with stable hemodynamics without significant bradycardia or hypotension. These findings support the safety of dexmedetomidine infusions for interfacility transport and provide a rationale for examining whether advanced life support clinicians can safely transport patients receiving dexmedetomidine infusions for this indication.

## INTRODUCTION

Over the past two years, there has been a significant increase in the potency of opioids as well as adulterants found in the illicit drug supply. One of the most common adulterants in the opioid supply in the Philadelphia region is medetomidine, an alpha-2-adrenergic agonist, similar to clonidine. Medetomidine was found in 88% of illicit opioids at the end of 2025 compared to 0% at the beginning of 2024.^1^ Adulterants such as medetomidine have transformed generally nonlife-threatening opioid withdrawal into a life-threatening condition manifesting as severe hemodynamic instability with marked hypertension and tachycardia. Dexmedetomidine, an alpha-2-adrenergic agonist, is used off-label to treat the life-threatening withdrawal from medetomidine because of its similar mechanism of action. This medication provides anxiolysis and sedation with minimal respiratory depression, making it an attractive option for treating this specific patient population.^2^,^3^

While the use of dexmedetomidine is well described in the emergency department (ED), intensive care unit (ICU), and procedural environments, data on its prehospital and interfacility setting use remain sparse.^4^,^5^ Use of dexmedetomidine in the prehospital environment is limited to case reports and small case series.^3^,^6^ This study evaluates the safety profile of dexmedetomidine infusion when used to treat complex opioid withdrawal during interfacility transport.

## METHODS

### Study Design

This is a retrospective case series of patients between January 1, 2025, and June 30, 2025, from a single interfacility transport agency. We included patients receiving dexmedetomidine infusions for presumed alpha-2-adrenergic agonist withdrawal. The agency provides both air and ground critical care interfacility transport in the Philadelphia region, running approximately 1,600 transports annually.

We identified charts using the internal reporting feature embedded in the electronic patient care reporting (ePCR) system. Search terms included “dexmedetomidine,” “Precedex,” “Dilaudid,” “hydromorphone.” The latter two terms were used as many patients who are transported on dexmedetomidine infusions are also transported on hydromorphone infusions. We used both generic and brand names, as well as opioid and alpha-agonist classifications, because of the absence of a standardized charting procedure. Additional search terms, including “withdrawal,” “opiate,” “opioid,” and “altered mental status,” were also applied but did not yield additional relevant cases. All identified charts were manually reviewed, and duplicate entries were consolidated into single cases. We excluded patients receiving only Dilaudid/hydromorphone or Precedex/dexmedetomidine for indications unrelated to complex withdrawal (eg, sedation adjunct for mechanical ventilation) because this study specifically focused on patients with alpha-2-agonist withdrawal. Data were extracted by a single reviewer using a standardized data extraction form. The analysis was performed by a separate author (SG).

**Population Health Research Capsule**

What do we already know about this issue?

*Medetomidine, an alpha-2 agonist adulterant in illicit opioids in the Philadelphia region, causes a life-threatening withdrawal syndrome*.

What was the research question?

*Does interfacility dexmedetomidine infusion cause clinically significant hemodynamic or airway instability?*

What was the major finding of the study?

*Dexmedetomidine use in the prehospital environment was safe with no episodes of hypotension/bradycardia or airway compromise*.

How does this improve population health?

*These findings support ALS transport of patients on dexmedetomidine for alpha-2-agonist withdrawal, potentially reducing transfer delays and improving ED throughput*.

This study was determined to be exempt by the Temple University Institutional Review Board.

### Data Collection

Extracted variables included age (where available), sex, vital signs, Glasgow Coma Scale (GCS) and dexmedetomidine infusion rate (mcg/kg/hr).

### Vital signs

Initial vital signs were defined as the first documented vital signs by the critical care transport team at the sending facility. All patients were started on a dexmedetomidine infusion prior to the arrival of the interfacility transport team and were maintained during transport at a level determined by the sending emergency physician. We defined destination vital signs as the final documented set at the time of transfer of care at the receiving facility. Vital sign measurements were obtained using standard interfacility monitoring equipment (Zoll X Series) (Zoll Medical Corporation, Chelmsford, MA) and documented according to agency protocol. To determine the maximum and minimum values, all vital signs obtained during transport were used. Dexmedetomidine dosing was analyzed as the documented continuous infusion rate in mcg/kg/hr. Bolus dosing of dexmedetomidine was not employed during transport.

### Outcome Definition

Our primary outcome measures were changes in systolic and diastolic blood pressure, mean arterial pressure (MAP), and heart rate from initial contact to destination. Secondary outcomes included the need for airway intervention. Hypotension was defined as MAP less than 65 mm Hg, or systolic blood pressure less than 90 mm Hg, at any time during transport. Bradycardia was defined as a heart rate less than 50 beats per minute (bpm), as this is our institutional guideline to decrease the dose of dexmedetomidine. Any interventions based on vital signs were noted. No predefined intervention thresholds were mandated by protocol.

### Statistical Analysis

We analyzed data using descriptive statistics where appropriate. Our primary outcome was evaluated using a paired *t*-test for continuous variables.

## RESULTS

A total of 217 patients were identified, with 182 patients having complete data for hemodynamic analysis (Table).

The average transport time was 15.7 minutes (range, 5–40 minutes). Infusion rates for dexmedetomidine ranged from 0.2 to 1.5 mcg/kg/hr, with a mean dose of 1.32 mcg/kg/hr (Figure 1).

### Hemodynamic Effects

Mean arterial pressure decreased from an average of 122.1 (21.7) mm Hg at the time of pickup to 118.3 (21.7) mm Hg at the drop-off destination (Figure 2). Mean heart rate showed minimal change (104.9 [23.7] bpm to 103.6 [21.8] bpm). The lowest recorded blood pressures and heart rates did not meet the definition of hypotension or bradycardia. One person received 500 mL of normal saline for a blood pressure reading of 99/59 with a MAP of 74 mm Hg.

## DISCUSSION

In this retrospective case series of patients receiving dexmedetomidine infusions for presumed alpha-2-agonist withdrawal during interfacility transports showed overall hemodynamic stability with mild reductions in MAP and minimal changes in heart rate. Importantly, no clinically significant episodes of hypotension or bradycardia requiring intervention were identified. The modest reduction in MAP observed is consistent with the known pharmacologic profile of dexmedetomidine and did not appear to translate into clinically significant adverse events. In addition, no patients required intubation, consistent with dexmedetomidine’s profile of maintaining respiratory neutrality. While the absence of a comparator group limits causal inference, the consistency of observed physiologic effects supports feasibility and relative safety in selected patients, especially patients who have increased sympathetic tone from acute withdrawal.

Currently, the interfacility transport of patients on dexmedetomidine infusions requires advanced level practitioners, at least at the level of a prehospital registered nurse (PHRN) and is not within the scope of advanced life support (ALS) clinicians in the Commonwealth of Pennsylvania. Because of current volume and staffing challenges, the need for a PHRN crew has resulted in significant delays in transferring patients between facilities to a higher level of care. These delays have led to increased ED boarding times and decreased patient throughput. Allowing ALS clinicians to transport this medication would significantly improve the movement of these patients to inpatient care environments and improve emergency department throughput.

## LIMITATIONS

This study is limited by its retrospective design, the inclusion of a single interfacility transport agency, and the absence of a control group. Patients also received a variety of medications prior to transport by the sending physician, including opioids, typical and atypical antipsychotics, ketamine, benzodiazepines, phenobarbital, and serotonin 5HT3 receptor antagonists. The measurements of vital signs were time-stamped in the ePCR, but there was not a standard time of when to obtain them upon arrival at the bedside.[Fig f3-wjem-27-1327]

## CONCLUSION

In this case series, interfacility dexmedetomidine infusions were associated with stable vital signs and no incidence of adverse hemodynamic or airway effects. The use of advanced clinicians for these transports may not be warranted and can potentially be accomplished by an ALS crew, saving resources, increasing availability of transport, and transferring patients more quickly to a higher level of care. This study provides the rationale for a prospective study of interfacility transport of dexmedetomidine infusions by ALS clinicians for alpha-2 agonist withdrawal.

## Figures and Tables

**Figure 1:**
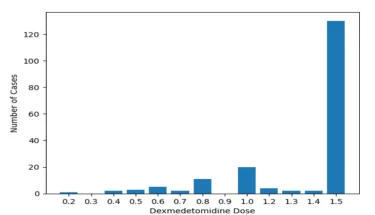
Dexmedetomidine dosing ranges (mcg/kg/hour) in a study describing the dosing and hemodynamic effects associated with the interfacility administration of dexmedetomidine in patients with alpha-2 adrenergic agonist withdrawal.

**Figure 2:**
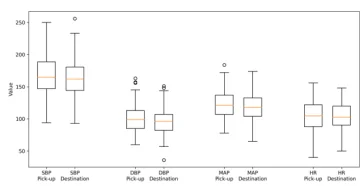
Hemodynamics at pickup and destination: Box plots comparing hemodynamic parameters at patient pickup and hospital arrival, including systolic blood pressure (SBP), diastolic blood pressure (DBP), mean arterial pressure (MAP), and heart rate (HR). Boxes represent interquartile ranges with median values; whiskers indicate the range of nonoutlier observations, and individual points represent outliers.

**Figure 3 f3-wjem-27-1327:**
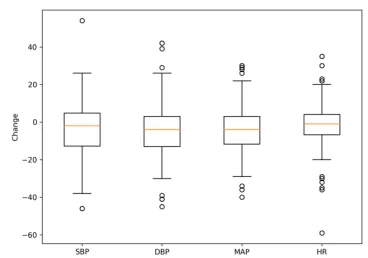
Change in hemodynamics (pickup to destination) during dexmedetomidine infusion**:** Box plots demonstrating change in systolic blood pressure (SBP), diastolic blood pressure (DBP), mean arterial pressure (MAP), and heart rate (HR) from patient pickup to hospital arrival following dexmedetomidine administration. Boxes represent interquartile ranges with median values; whiskers indicate the range of nonoutlier observations, and individual points represent outliers.

**Table:** Patient demographics and clinical characteristics in a study describing the dosing and hemodynamic effects associated with the interfacility administration of dexmedetomidine in patients with alpha-2 adrenergic agonist withdrawal.

Number of patients, *n*	182
Age, years	
Mean (SD)	39.5 (9.0)
Median (IQR)	39 (34–44)
Male sex, *n* (%)	121 (66.5%)
GCS at time of EMS pickup, median (IQR)	13 (10–15)
Dexmedetomidine dose (mcg/kg/hr), median (IQR)	1.5 (1.2–1.5)

*EMS*, emergency medical service; *GCS*, Glasgow Coma Scale; *IQR*, interquartile range; *SD*, standard deviation.
